# Chitosan oligosaccharide induces resistance to *Tobacco* mosaic virus in Arabidopsis via the salicylic acid-mediated signalling pathway

**DOI:** 10.1038/srep26144

**Published:** 2016-05-18

**Authors:** Xiaochen Jia, Qingshan Meng, Haihong Zeng, Wenxia Wang, Heng Yin

**Affiliations:** 1 Liaoning Provincial Key Laboratory of Carbohydrates, Dalian Institute of Chemical Physics, Chinese Academy of Sciences, Dalian 116023, China; 2 University of Chinese Academy of Sciences, Beijing, 100049, China; 3 College of Life Science, Dalian University of Technology, Dalian 116023, China; 4 College of Food Science and Engineering, Dalian Ocean University, Dalian 116023, China

## Abstract

Chitosan is one of the most abundant carbohydrate biopolymers in the world, and chitosan oligosaccharide (COS), which is prepared from chitosan, is a plant immunity regulator. The present study aimed to validate the effect of COS on inducing resistance to *tobacco mosaic virus* (TMV) in *Arabidopsis* and to investigate the potential defence-related signalling pathways involved. Optimal conditions for the induction of TMV resistance in *Arabidopsis* were COS pretreatment at 50 mg/L for 1 day prior to inoculation with TMV. Multilevel indices, including phenotype data, and TMV coat protein expression, revealed that COS induced TMV resistance in wild-type and jasmonic acid pathway- deficient *(jar1*) *Arabidopsis* plants, but not in salicylic acid pathway deficient (NahG*) Arabidopsis* plants. Quantitative-PCR and analysis of phytohormone levels confirmed that COS pretreatment enhanced the expression of the defence-related gene PR1, which is a marker of salicylic acid signalling pathway, and increased the amount of salicylic acid in WT and *jar1*, but not in NahG plants. Taken together, these results confirm that COS induces TMV resistance in *Arabidopsis* via activation of the salicylic acid signalling pathway.

Chitin is an abundant, water insoluble biopolymer, which is found mainly in the hard outer skeleton of marine animals, as well as in mushrooms and yeasts^1^,^2^. Chitosan is the product of chitin deacetylation and has limited application due to its insolubility in both organic solvents and water. Chitosan oligosaccharide (COS), derived from the enzymatic hydrolysis of chitosan, overcomes this limitation^1^,^3^. To date, COS has been shown to have a wide range of biological applications, including use as a health food^4^, a plant growth stimulator^5^,^6^, feed additive^7^, and an antimicrobial agent^8^.

The most noteworthy application of COS is its use as a regulator of plant immunity^9^. In 1980, Hadwiger first reported that COS could induce plant immunity^10^. Since then, COS has been considered as a potent elicitor of plant immunity that is used in many plants, including tobacco^11^, camellia^6^, wheat^12^, oilseed rape^13^, tomato^14^, soybean^15^, and grapevine^16^,^17^. However, the mechanism of COS-induced immunity in plants, especially the signalling processes involved, remains unclear.

Salicylic acid (SA) and jasmonic acid (JA) are two plant hormones essential for defence signal transduction^18^,^19^,^20^, which signal through two different pathways. SA mediates systemic acquired resistance (SAR), while JA mediates induced systemic resistance (ISR). SA and JA signalling pathways influence each other through a complex network of synergistic and antagonistic interactions^19^,^21^.

The results of previous studies have shown that COS induces the production of plant hormones, especially JA, in plants. Following COS treatment, the JA content in tomato^22^, rice^23^, and oilseed rape^13^ increased, suggesting that COS activates plant immunity through the JA signalling pathway in some plant systems. However, other studies have reported the reverse effect. For example, Obara *et al*. found that methyl-salicylic acid (MeSA) accumulates in rice leaves 7 h after COS treatment^24^.

Tobacco mosaic virus (TMV) is a plant virus used to study plant disease, and SA and JA signalling pathways have both been shown to be involved in plant defence following TMV infection. In tobacco and tomato, SA treatment was found to increase resistance to TMV, which resulted in reduced viral accumulation and delayed appearance of disease symptoms^25^,^26^. In addition, exogenous application of jasmonic acid methyl ester reduced local resistance to TMV and permitted systemic viral movement^27^. However, other reports on TMV-infected tobacco, showed that the expression of genes associated with the JA-mediated defence pathway were significantly upregulated after 1 d, whereas genes associated with the SA-mediated defence pathway were significantly upregulated after 5 days. Thus, both SA and JA are required for systemic resistance against TMV in tobacco^28^. We previously confirmed that COS could induce resistance to TMV in plants^9^,^29^,^30^; however, the signalling pathway involved in this resistance is unknown.

Mutants with defective signalling pathways are important experimental models, especially in the study of SA and JA in plant resistance^31^,^32^,^33^. *Jar1*, an ethyl methane sulfonate mutant first obtained by Staswick *et al*., contains a mutation in AT2G46370^31^, which encodes jasmonic acid-amido synthetase JAR1. *Jar1* plants have reduced sensitivity to JA, and have been widely used as a JA signalling-deficient mutant since 1992^33^,^34^,^35^,^36^. NahG plants, in which the bacterial nahG gene has been introduced, encode salicylate hydroxylase, which degrades SA. These plants accumulate very little SA and therefore have a defective SA pathway. This mutant was first introduced by Delaney *et al*. in 1994, and has been widely used in studies of plant defence and signalling^37^,^38^,^39^,^40^,^41^.

In the present study, multilevel indices, including phenotype data, TMV coat protein (TMV-CP) gene expression, and TMV-CP levels were investigated to confirm the effect of COS on inducing resistance to TMV in *Arabidopsis*. Furthermore, *jar1* and NahG *Arabidopsis* mutants were employed to explore the signalling pathways involved in COS-induced resistance to TMV.

## Results

### COS induces resistance to TMV in *Arabidopsis*

**Figure 1:**
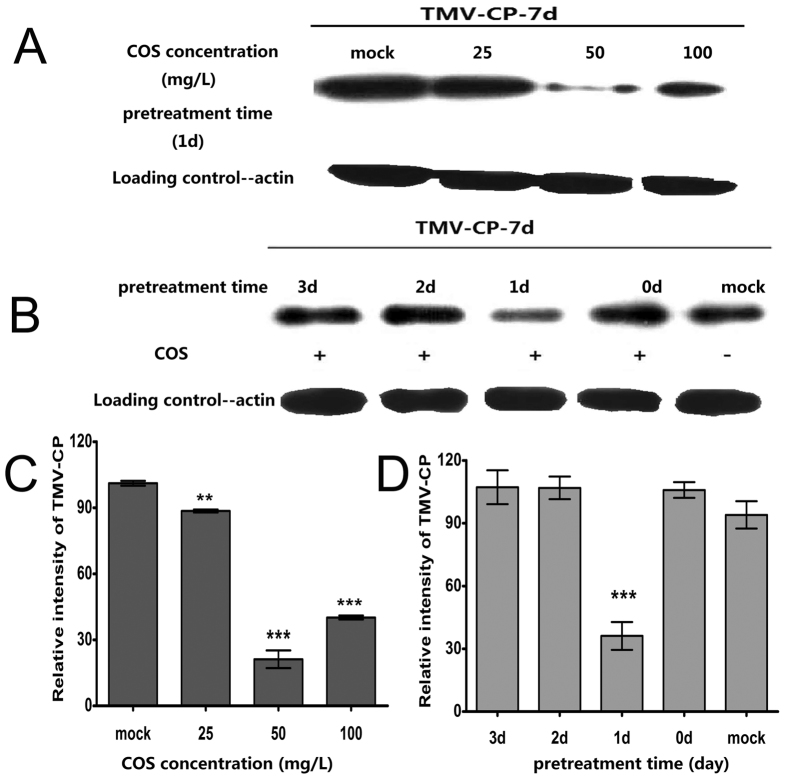
The most effective COS concentration and pretreatment time. TMV-CP was detected by western-blot using anti-TMV antibody.(**A**) The amount of TMV-CP in leaves pretreated 1d before *TMV* inoculation with different concentrations of COS. (**B**) The amount of TMV-CP in leaves pretreated with 50 mg/L COS with different pretreatment time. All samples were inoculated *TMV* for 7d, more than 30 *Arabidopsis* plants were used in each groups. These were a representative experiment and were independently repeated three times using different samples from independent treatment. The same amount of protein was loaded on SDS-PAGE gels, detected by anti-plant actin antibody, which served as the protein loading control. Full-length blots were uploaded as supplementary information (Supplementary Fig. A,B). The detection of TMV-CP and loading control were carried out under the same experimental conditions. (**C**,**D**) The relative TMV-CP levels in (**A**) and (**B**) were quantified by scanning densitometry using image j program. The quantitative densitometry values (histograms) which normalized to the loading controls were expressed as mean ± SD of three independent experiments. Asterisks indicate significant differences (**P < 0.01; ***P < 0.001).

The most effective concentration of COS, and the optimal pretreatment time, for resistance to TMV were studied in wild *Arabidopsis* plants. The TMV-CP levels in inoculated leaves showed that COS could induce TMV resistance in *Arabidopsis* (Fig. 1). The most effective COS concentration was 50 mg/L (Fig. 1A,C) and the optimal pretreatment time was 1 day (Fig. 1B,D).

### The effectiveness of COS on defence signalling pathway mutants

In order to explore the signalling pathways involved in COS-induced resistance to TMV in *Arabidopsis*, WT, JA signalling-deficient mutant (*jar1*) and SA signalling-deficient mutant (NahG, *sid2*) plants were used.

**Figure 2:**
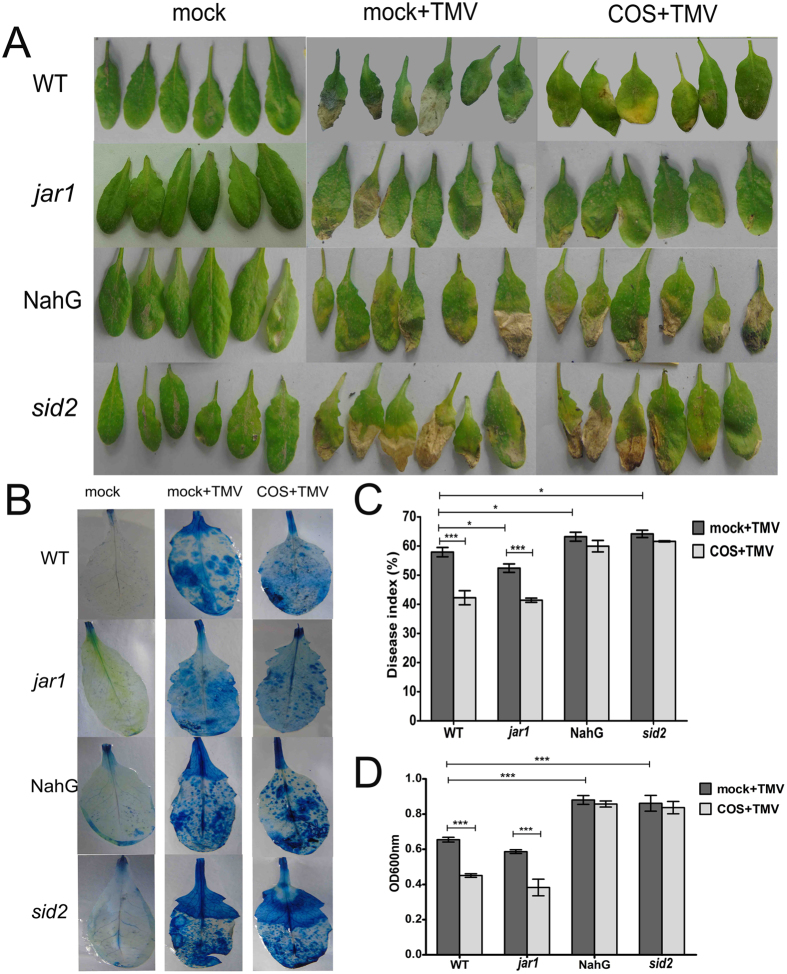
The effect of COS treatment on different mutants. Mock means pretreated *Arabidopsis* with water, and then inoculated with PBS by gently rubbing the leaf surface using Carborundum (silicon carbide). Mock + TMV or COS + TMV means pretreated *Arabidopsis* with water or 50 mg/L COS, and then inoculated with *TMV* by gently rubbing the leaf surface using Carborundum (silicon carbide). (**A**) The necrotic lesion on leaves after inoculated with *TMV* for 7d in different mutants. (**B**) The amount of cell death in rosette leaves of *Arabidopsis* which inoculation with *TMV* 7d was stained with Evans blue. (**C**) The disease index which evaluated the necrotic lesion on leaves of different mutants. Values are the means ± SD from three independent measurements. Asterisks indicate significant differences (*P < 0.05, **P < 0.01; ***P < 0.001). (**D**) The amount of cell death in rosette leaves, by measured the absorption under 600 nm using spectrophotometer. Values are the means ± SD from three independent measurements. Asterisks indicate significant differences (**P < 0.01, ***P < 0.001).

The symptoms of TMV infected leaves showed multiple necrotic lesions (Fig. 2A). The areas of necrotic lesions on infected leaves were evaluated (Fig. 2B). The symptoms on NahG leaves (63.2% ± 2.7%) and *sid2* (64.2% ± 2.2%) were more severe than those on WT (57.8% ± 2.7%) and *jar1* (52.4% ± 2.5%) leaves after 7-days TMV infection (Fig. 2B). In COS-pretreated plants, the TMV symptoms on WT (42.2 ± 4.2%) and *jar1* (41.4% ± 1.3%) leaves were less severe, but no significant effect was observed on NahG (61.0% ± 3.4%) and leaves *sid2* (61.6% ± 0.3%). These results showed that COS could induce resistance to TMV in *Arabidopsis*, and that COS was more effective in WT and *jar1* plants.

Cell death in the rosette leaves of *Arabidopsis* following a 7-day inoculation with TMV was observed by Evans blue staining. Increased levels of cell death were observed in NahG leaves than in WT and *jar1* leaves. Cell death in WT and *jar1* leaves was not observed in the COS pretreated group, but little effect was observed in NahG and *sid2* plants (Fig. 2C,D). These data suggested that COS pretreatment could inhibit cell death in leaves during TMV infection in a SA-dependent manner.

**Figure 3:**
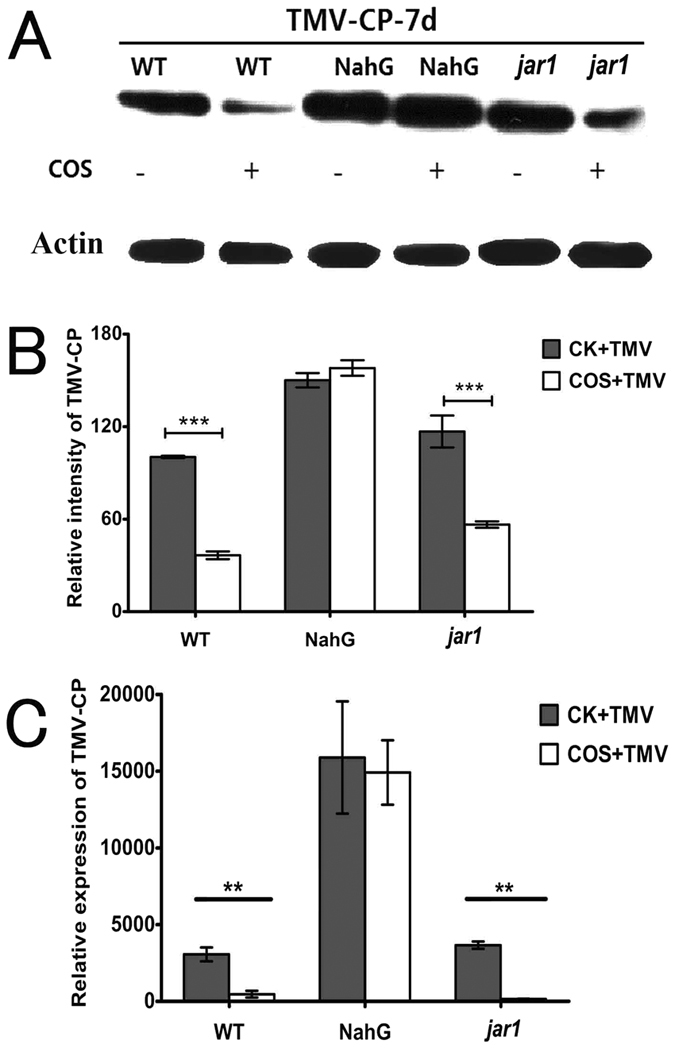
The expression of TMV-CP in rosette leaves of different *Arabidopsis* mutants. (**A**) The amount of TMV-CP in leaves was detected by western-blot. All samples were inoculated TMV for 7d, more than 30 *Arabidopsis* plants were used in each groups. This was a representative experiment and was independently repeated three times using different samples from independent treatment. The same amount of protein was loaded on SDS-PAGE gels, detected by anti-plant actin antibody, which served as the protein loading control. Full-length blots were uploaded as supplementary information (Supplementary Fig. C). The detection of TMV-CP and loading control were carried out under the same experimental conditions. (**B**) The relative TMV-CP levels in (**A**), which was quantified by scanning densitometry using image j program. The quantitative densitometry values (histograms) which normalized to the loading controls were expressed as mean ± SD of three independent experiments. Asterisks indicate significant differences (***P < 0.001). (**C**) The expression of TMV-CP in leaves was detected by q-PCR, using *actin8* as reference gene. Values were presented as the means ± SD from three independent measurements. Asterisks indicate significant differences (**P < 0.01).

In addition, mRNA and proteins levels of TMV-CP were determined in inoculated leaves. The q-PCR (Fig. 3C) and western blot data (Fig. 3A,B) showed that the expression of TMV-CP mRNA and protein in NahG leaves was much higher than that in WT and *jar1* leaves. In COS pretreated plants, the expression of TMV-CP mRNA and protein was markedly decreased in WT and *jar1* leaves, but was not obviously changed in NahG leaves.

Taken together, these data confirm that COS pretreatment induced resistance to TMV in WT and *jar1* plants, but did not have an obvious effect in NahG plants, implying that COS induces TMV resistance via the SA signalling pathway.

### Expression of defence-related genes

To explore the role of SA and JA signalling pathways in COS-induced resistance to TMV, we examined the expression of signalling pathway marker genes, including PR1 (a SA pathway marker) and PDF1.2 (a JA pathway marker) in leaves following infection with *TMV* for 7 days.

**Figure 4:**
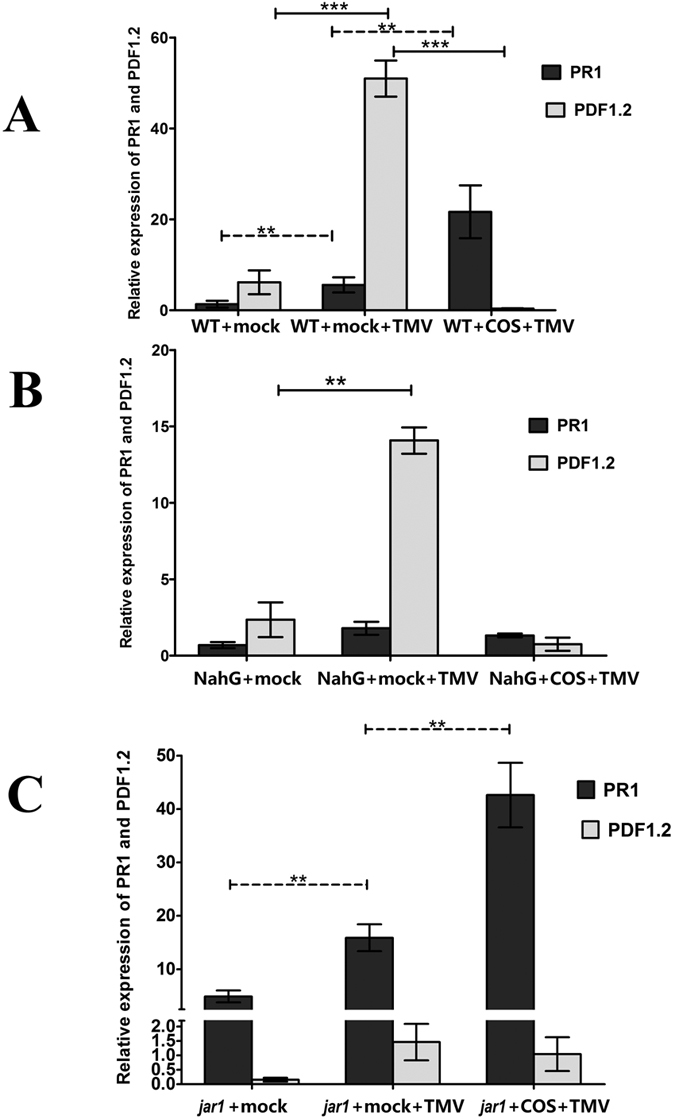
The relative expression of PR1 and PDF1.2 in different mutants detected by q-PCR. Mock + TMV or COS + TMV means *Arabidopsis* pretreated with water or 50 mg/L COS, then inoculated with *TMV* by gently rubbing the leaf surface using Carborundum (silicon carbide). Mock means pretreated *Arabidopsis* with water, then inoculated with PBS. (**A**) The expression of PR1 and PDF1.2 in WT. (**B**) The expression of PR1 and PDF1.2 in NahG mutant. (**C**) The expression of PR1 and PDF1.2 in *jar1* mutant. All data were measured using *actin8* as reference gene. Values were presented as means ± SD from three independent measurements. Asterisks indicate significant differences (**P < 0.01; ***P < 0.001).

In WT *Arabidopsis*, TMV infection (WT + mock + TMV) promoted a 4.16-, and 8.28-fold increase in the expression of PR1 and PDF1.2, respectively, compared with the control group (WT + mock) (Fig. 4A). Following COS pretreatment (WT + COS + TMV), PDF1.2 expression was inhibited but the expression of PR1 was increased 5.37-fold compared with that observed in the WT + mock + TMV group.

In NahG plants, which have a defective SA signalling pathway, the expression of PR1 (NahG + mock) was much lower compared with that in the related WT control (2.09-fold lower, WT + mock) and *jar1* (7.10-fold lower, *jar1* + mock) groups. In NahG plants, TMV infection (NahG + mock + TMV) resulted in up-regulation of PDF1.2, which was increased 5.99-fold compared with the control (NahG + mock) (Fig. 4B). In the COS pretreated group (NahG + COS + TMV), PR1 expression was not enhanced compared with that in the WT and *jar1* plants, since SA signalling is defective in NahG plants; however, the expression of PDF1.2 was still inhibited (Fig. 4B).

In *jar1* plants, which have defective JA signalling, the level of PDF1.2 expression was much lower compared with the WT and NahG plants. The expression of PR1 in *jar1* plants (*jar1* + mock) was higher than that in WT plants (3.66-fold increase, WT + mock), while the expression of PDF1.2 was decreased 39.61-fold. In *jar1* mutant plants, TMV infection (*jar1* + mock + TMV) resulted in the upregulation of PR1 expression, which increased 2.55-fold compared to the control plants (*jar1* + mock) (Fig. 4C). In the COS pretreated group (*jar1* + COS + TMV), the expression of PR1 was enhanced 7.31-fold compared to the control group (*jar1* + mock) (Fig. 4C).

In conclusion, expression of the signalling pathway defence marker PR1 was upregulated following COS pretreatment in WT and *jar1* plants, which suggests that COS-induced TMV-resistance in *Arabidopsis* is mainly dependent on the SA signalling pathway.

### COS induced changes in SA and JA level in *Arabidopsis*

**Figure 5:**
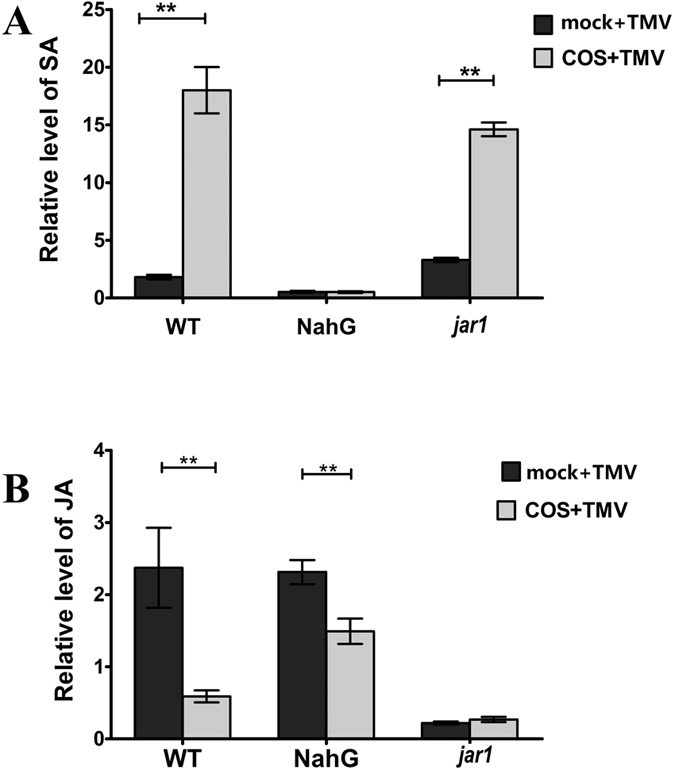
The relative level of SA and JA in different mutants. mock + TMV or COS + TMV means *Arabidopsis* pretreated with water or 50 mg/L COS, then inoculated with *TMV* by gently rubbing the leaf surface using Carborundum (silicon carbide). (**A**) The content of SA in different mutants. (**B**) The JA accumulation in different mutants. WT + mock were used as the control group. Values were presented as means ± SD from three independent measurements. Asterisks indicate significant differences (**P < 0.01).

To further confirm that COS induces resistance to TMV in *Arabidopsis* via the SA signalling pathway, the levels of SA and JA were determined in inoculated leaves. The SA content was clearly enhanced in COS-pretreated WT (WT + COS + TMV) and *jar1* (*jar1* + COS + TMV) plants, with increases of 10.01- and 4.43-fold observed, respectively, compared with the mock + TMV groups (WT + mock + TMV, *jar1* + mock + TMV) (Fig. 5A), which is consistent with the expression of PR1 shown in Fig. 4. The SA content in NahG plants was much lower than that in the related WT and *jar1* plants, and COS pretreatment had no effect on expression in NahG plants (Fig. 5A).

TMV infection increased the JA content in WT and NahG plants, whereas in COS pretreated plants, the JA content was decreased due to an increase in the level of SA (Fig. 5B). The JA content in *jar1* was much lower than that in WT and NahG (Fig. 5B) plants, since the jasmonic acid-amido synthetase JAR1 was dysfunctional in *jar1* mutants. Phytohormone data suggested that COS induces resistance to TMV in *Arabidopsis*, in a manner that is dependent on the SA signalling pathway.

### COS induced NO production in *Arabidopsis* rosette leaves

**Figure 6:**
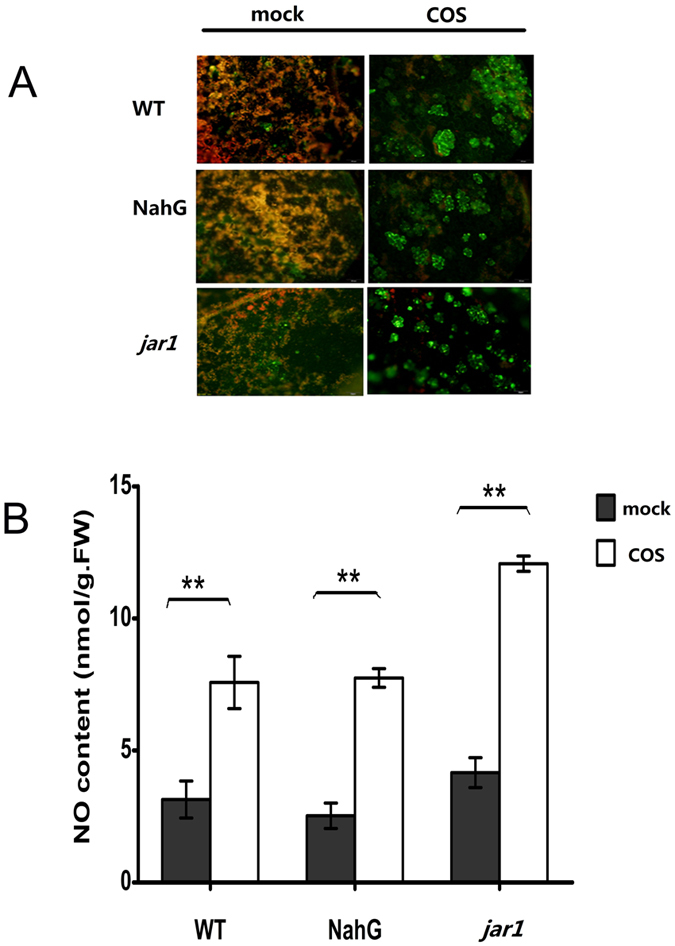
The NO production in rosette leaves of *Arabidopsis*. (**A**) The NO production (green) in epidermal cells of different *Arabidopsis* mutants after COS treatment, observed by using fluorescence microscopy. (**B**) The NO content in rosette leaves measured by Griess reagent. Values are the means ± SD from three independent measurements. Asterisks indicate significant differences (**P < 0.01).

NO is an important signalling messenger involved in plant immunity. We found that COS induced NO production in epidermal cells in WT, NahG, and *jar1* mutant plants (Fig. 6A). To further quantify changes in NO production following COS treatment in different mutants, the NO content in leaves was measured. After the rosette leaves were sprayed with 50 mg/L COS, the NO content in leaves of different mutants (WT, NahG, *jar1*) increased 2.41-, 3.06-, 2.90-fold, respectively, compared to that in the mock-treated control group.

In conclusion, COS induced NO production in *Arabidopsis* leaves independent of the SA and JA signalling pathways.

## Discussion

COS has been shown to elicit plant defence responses to a broad spectrum of pathogens in many plant species, including tobacco, rapeseed, rice, and grapevine^11^,^16^,^30^,^42^,^43^. The duration and concentration of pretreatment are important for the effect of COS on plant defence. In a previous study, plants pretreated with COS prior to pathogen infection were get better control efficacy than COS treatment after pathogen infection^9^. The optimal duration of COS pretreatment differs for different plant-pathogen interactions. For example, the optimal duration of COS pretreatment is 1 day for tobacco-TMV interactions^29^, and 3 days for rapeseed*-S. sclerotiorum* interactions^44^. The reason for this difference may be because COS signal transduction, and the plant response to different diseases occur differently in different plants^45^. As a broad-spectrum plant elicitor, low concentrations of COS (25–100 mg/L) were most effective at activating plant disease defences^12^,^46^. Thus, in the present study, the optimum pretreatment time (0–3 days) and concentration (0–100 mg/L) were explored using TMV-CP protein levels as the disease evaluation index. We found that COS could induce TMV resistance in *Arabidopsis* and that the most effective concentration was 50 mg/L with a pretreatment duration of 1 day prior to inoculation. These data confirm that COS is an effective elicitor of plant immunity in TMV-infected *Arabidopsis*.

The hypersensitive response (HR) occurs during induced disease resistance in plants. HR is characterized by rapid cell death at the site of pathogen invasion, commonly resulting in the formation of necrotic lesions in which the pathogen is thought to localize, thereby inhibiting its multiplication and spread^47^. Thus, the area of the necrotic lesion correlates with the amount of virus present on the leaves, to some extent. Data obtained in the present study regarding phenotype data (Fig. 2) were consistent with those obtained by q-PCR (Fig. 3C) and western blotting (Fig. 3A,B) for TMV-CP in rubbed leaves. These data confirm that COS induced TMV resistance in *Arabidopsis*, which was more obvious in WT and *jar1* plants. We used these four data from different levels to get this conclusion, so this research work was more systemic and convinced than former works^5^,^6^,^13^,^17^. Taken together, the results of the present study confirm that COS induces TMV resistance to *Arabidopsis* via the SA signalling pathway.

The results of a previous study suggested that SA plays a critical role in plant defence against virus invasion. In compatible host-virus interactions, SA reduces viral replication and restricts cell-to-cell and systemic movement, and several defence-related genes are induced by SA-dependent signalling^48^,^49^. Exogenously applied SA increased resistance to TMV in tomato, and TMV infection increased the endogenous SA content^26^. Activation of SA biosynthesis or signalling in many plant-pathogen interactions has been shown to inhibit JA biosynthesis or signalling^21^,^50^. Exogenously applied methyl jasmonate, a methyl ester of JA, reduced local resistance to TMV and permitted systemic viral movement in tobacco plants^27^. Thus, the results of previous studies suggested that SA functions as a positive regulator of TMV resistance. This conclusion is consistent with our findings that COS induces TMV resistance in *Arabidopsis* by upregulating the expression of PR1 and increasing the level of SA.

Previous studies have shown that several genes associated with JA synthesis and regulation are induced by COS in *Brassica napus*^45^ and that the JA content in tomato^22^, rice^23^, and rapeseed^13^ is increased. Those results suggested that COS defence signalling is mainly mediated by activation of the JA signal pathway under some plant-disease interactions. However, the results of the present study showed that COS activated the SA signalling pathway, suggesting that COS induces plant defences via different signalling pathways during different plant-disease interactions. This is a common phenomenon for many plant elicitors.

For example, harpin, the product of the *hrpN* gene of *Erwinia amylovora*, is an effective elicitor that induces plant responses in many plants^51^,^52^,^53^. Harpin induced disease (*Peronospora parasitica* and *Pseudomonas syringae*) resistance in *Arabidopsis* through activation of the SA signalling pathway^54^. However, in harpin-treated *Phalaenopsis* orchids, JA signalling was activated^55^. This phenomenon was also found for flg22, another effective elicitor that induces plant defence responses. Flg22 treatment induced SA accumulation and increased the expression of typical SA-related genes^56^,^57^ in *Arabidopsis*. Flg22-triggered oxidative bursts have been shown to depend on SA signalling^58^. In addition, flg22 induced *Arabidopsis* resistance to *Golovinomyces cichoracearum* through SA-mediated signalling^59^. However, analysis of *Arabidopsis* mutants showed that JA, ethylene, and SA signalling all contribute positively to flg22-triggered immunity to a virulent bacterial strain, *Pseudomonas syringae DC3000*^60^.

Those results suggested that the type of plant disease is important for the elicitor to activate a specific defence signal pathway. Usually, biotrophic and hemi-biotrophic pathogens are more sensitive to SA-dependent responses, whereas necrotrophic pathogens and herbivorous insects are commonly resisted by JA-dependent defence mechanisms^61^,^62^. Elicitors signal through appropriate signalling pathways in order to resist different diseases; thus, the effect of the elicitor becomes broad spectrum.

The q-PCR data presented in this study (Fig. 4) showed that the expression of PR1 was higher in *jar1* plants compared with WT plants (3.66-fold increase), and COS enhanced PR1 expression to a greater level in *jar1* mutants (1.93-fold increase), implying that the resistance to TMV was stronger in *jar1* plants. Indeed, from the disease index (Fig. 2C), the effect of COS on TMV disease control was more obvious in *jar1* plants. However, from the cell death level (Fig. 2D) and the expression of TMV-CP (Fig. 3), the disease control in *jar1* was not enhanced compared with WT (no significant difference). Furthermore, the SA content (Fig. 5A) was not completely consistent with the expression of PR1 (Fig. 4). Signalling networks in plants are highly complex; therefore, further research is needed.

Once COS signals reach the cytoplasm, they activate a complicated signalling network, which requires further study. The results of the present study suggest that COS induces TMV resistance in *Arabidopsis*, mainly through the SA signalling pathway. Whether an early signalling molecule, such as NO is involved in COS-induced resistance, and whether it acts in a SA-dependent manner, remains unknown. To address this, we measured NO production in different *Arabidopsis* mutants following COS pretreatment. In a previous study, COS promoted NO production in epidermal cells of *B. napus* leaves shortly after treatment. NO was generated within 5 min of treatment, lasted for more than 30 min, and declined after 2 h^13^. We found that the NO content in rosette leaves of different *Arabidopsis* plants (WT, NahG, *jar1*) increased following 15-min COS treatment (Fig. 6). Those results suggested that COS induced NO production in epidermal cells of *Arabidopsis* in a SA- and JA-independent manner, implying that NO may be acting upstream of the SA and JA signalling pathways.

In conclusion, COS induces resistance to TMV in *Arabidopsis* at an optimal pretreatment dose of 50 mg/L for 1 day before inoculation with TMV. Furthermore, from the phenotype, TMV-CP expression, defence gene expression, and phytohormone results, we confirm that COS induces TMV resistance via the SA signalling pathway. From this and previous findings, we conclude that COS induces different signalling pathways involved in plant defence according to the type of plant disease.

## Materials and Methods

### Reagents

COS with a degree of polymerization (DP) from 2 to 10 and a degree of deacetylation (DD) of 95% was obtained from Dalian GlycoBio (Dalian, China). Reagents for use in molecular biology studies were purchased from TaKaRa Biotechnology (Dalian, China). SA and JA standards were obtained from sigma. The remaining chemicals were all of analytical grade and were purchased from Chinese companies.

### Plant materials and treatment

*Arabidopsis* ecotype Columbia (WT) and mutants including *jar1* (JA-defective mutant) and NahG (express the bacterial *nahG* gene encoding salicylate hydroxylase to degradation SA), *sid2* (SA-deficient mutant, a generous gift from Dr. Lin Hao’s Laboratory, Shenyang Normal University) were used in this experiment. The seed germination medium was composed of half-strength MS basal salts, with 1% sucrose and 0.8% agar, pH 5.8. All of the seeds were sterilized and planted on the agar plates. After cold treatment, the plates were oriented vertically in the growth chamber under 12 h of light, 12 h of dark, 22 °C, 55% humidity, with a light intensity of ~120 μmol m^−2^s^−1^ for germination. The seedlings were transplanted in plastic pots containing a mixture of vermiculite and sterilized loamy soil (1:1, v/v) on the 7th day after germination, then grown in the growth chamber for 30 days, before being used in this experiment.

To determine the most effective treatment method, WT plants were pretreated by spraying different concentrations of COS (0, 25, 50, 100 mg/L) one day before inoculation with TMV to search for the most effective COS concentration. WT plants were then pretreated with COS for different durations (3, 2, 1, 0 day), and then inoculated with *TMV* to identify the best pretreatment time. Each group contained more than 30 *Arabidopsis* plants, which were divided equally between three parallel experiments. The experiments were repeated at least three times.

To identify the major signalling pathway involved in COS-induced resistance to TMV, the WT, NahG, *sid2* and *jar1* plants were evenly assigned into control group (mock) (sprayed with water 1 day before inoculation with 50 mM phosphate buffer (PBS)), COS group (sprayed with COS 1 day before inoculation with PBS), mock + TMV group (sprayed with water 1 day before inoculation with TMV), COS + TMV group (sprayed with COS 1 day before inoculation with TMV). Each group contained more than 30 *Arabidopsis* plants, divided equally into three parallel experiments. The experiments were repeated at least three times.

### TMV inoculation

TMV was kept in our lab and multiplied in tobacco. TMV was extracted from infected tobacco leaves by homogenization in 50 mM phosphate buffer (pH 5.5) (1 g/5 mL), then centrifuged at 2000 × *g* for 6 min. The supernatant extract was used for mechanical inoculation^29^.

Rosette leaves of *Arabidopsis* plants were used for TMV inoculation. Inoculation was performed by gently rubbing the leaf surface using carborundum (silicon carbide) with viral suspension or 50 mM phosphate buffer (pH 5.5). Plants were then inoculated at 22 °C with 12 h of light at 120 μmol m^−2^ s^−1^.

Symptoms were assessed 7 days after TMV infection as follows: first, counting the leaves number in each level (the percentage of necrotic lesions areas on leaves(S)): 0 < S < 0.25, 1 level; 0.25 < S < 0.5, 2 level; 0.5 < S < 0.75, 3 level; 0.75 < S < 1, 4 level. Then, the following formula was used to obtain symptom data: disease index (%) = (∑level × leaves per level/total number of leaves × the highest level) × 100. Inoculated leaves were sampled 7 days after TMV infection, weighed, frozen in liquid nitrogen, and kept in −80 °C until use.

### SDS-PAGE and immunoblotting

Plant material was ground in liquid nitrogen, resuspended in 200 μL 20 mM PBS (20 mM Na_2_HPO_4_, and 20 mM NaH_2_PO_4_, pH 7.4) per 100 mg of plant material in an ice bath, and centrifuged. Part of the supernatant was used to determine protein concentration, by the Bradford method using bovine serum albumin as a standard (Bradford 1976). The remaining supernatant was immediately mixed with SDS-PAGE loading buffer, denatured in boiling water for 5 min, and the same amount of protein was subjected to two SDS-PAGE (10%) gels. Western blotting was performed using polyvinylidene difluoride membranes, blocked with 5% (w/v) non-fat dry milk in Tris-buffered saline (20 mM Tris-HCl, pH 8.0, and 150 mM NaCl) with 0.05% Tween 20.

One membrane was probed with an anti-TMV-CP antibody (1:3000; a generous gift from Dr. Huaifang Li’s Laboratory, China Agricultural University). Bound primary antibody was detected with ECL reagent after incubation with a goat anti-rabbit antibody (1:2000; Santa Cruz). Another membrane was probed with an anti-plant actin antibody (1:1500; Earthox), and goat anti-mouse antibody (1:2000; Earthox) as a second antibody. Actin was detected under the same conditions, and served as a protein loading control. Each data point was normalized against the corresponding actin data point. To obtain quantitative results, immunoblots were analysed by densitometry using Image J software (National Institutes of Health, Bethesda, MD, USA).

### Measurement of cell death

Evans blue staining was used as a marker of cell death, as follows. The leaves incubated with TMV for 7 days were soaking in 0.05% Evans blue for 24 h, washed with water, dried with filter paper, and then soaked in boiled anhydrous alcohol and glycerol (9:1 V/V) for 30 min to remove the chlorophyll. The leaves were imaged with a digital camera, and the blue areas on leaves represent cell death^63^.

To estimate the level of cell death in *Arabidopsis thaliana* leaves caused *by TMV* infection, the leave tissues that had been stained with Evans blue were added to 4 mL 1% SDS water solution to extract Evans blue for 3 days. The tubes were centrifuged at 9000 × *g* for 3 min. The supernatant was removed and the optical density was determined at 600 nm using a spectrophotometer^64^.

### Assay of nitric oxide production

NO production was detected using the fluorescent indicators DAF-2DA^13^ with some modification. The epidermis was peeled carefully from the surface of rosette leaves and then incubated in MES/KCl buffer (10 mM MES/KOH, 50 mM KCl, 100 μM CaCl_2_, pH 6.5) for 30 min in the light. Then, the epidermis was incubated in Tris/KCl buffer (10 mM Tris, 50 mM KCl, pH 7.2) containing 5 μmol/L DAF-2DA (sigma) for 30 min at room temperature in the dark. After excess DAF-2DA was removed by washing three times with fresh Tris/KCl buffer, the epidermis was placed in Tris/KCl buffer containing COS for 15 min, Tris/KCl buffer as contrast. NO production was detected using fluorescence microscopy with an excitation wavelength of 430–485 nm and an emitting wavelength of 515 nm.

The NO content in leaves of different *Arabidopsis* mutants was determined using Griess reagent (Beyotime Biotech) according to the manufacturer’s instructions. Rosette leaves of WT, NahG, and *jar1* plants were sprayed with COS or water, incubated for 15 min, picked and weighed, then quickly frozen in liquid nitrogen for subsequent NO content determination. The experiments were repeated at least three times for each treatment.

### Quantitative PCR (q-PCR) Analysis of Gene Expression

Total RNA for q-PCR analysis was extracted using TRIzol reagent (Invitrogen, Carlsbad, CA, USA) according to the manufacturer’s instructions, and then quantified by ScanDrop100 (AnalytikJenaAG, German). RNA of the same quality was reverse transcribed into cDNA with AMV Reverse Transcriptase (Takara). Dilutions of cDNA were used as templates in q-PCR (qTOWER 2.2, AnalytikJenaAG, German) using a SYBR Green kit (Bio-Rad) with gene-specific primers designed using Premier 5.0 (Table 1). Relative expression levels were calculated using the **2**^**−ΔΔCt**^ method^65^, and were all normalized to the expression of actin in each group.

### SA and JA Measurements

Quantification of JA and SA in inoculated *Arabidopsis* leaves was performed using a liquid chromatography-electrospray ionisation tandem mass spectrometry method as previously described^66^ with some modification. Chromatography conditions were modified by using a C18 column (Thermo, 250 × 4.6 mm), and an elution gradient of 5 ml/L formic acid (A) and acetonitrile (B) was set at a flow rate of 1 mL/min. Elution program (t min [%A:%B]) was: 0 min (90:10), 25 min (20:80), 26 min (90:10), and 35 min (90:10). The temperature of the column oven was 30 °C and the injection volume was 10 μL. Mass spectrometry conditions were modified by using Q-trap 5500 (AB SCIEX, USA) with different collision energy.

## Additional Information

**How to cite this article**: Jia, X. *et al*. Chitosan oligosaccharide induces resistance to *Tobacco mosaic virus* in *Arabidopsis* via the salicylic acid-mediated signalling pathway. *Sci. Rep*. **6**, 26144; doi: 10.1038/srep26144 (2016).

## Supplementary Material

Supplementary Information

## Figures and Tables

**Table 1:** Primers used for quantitative PCR.

Gene names	Locus ID	Forward	Reverse
*Atactin8*	AT1G49240	CACATGCTATCCTCCGTCTC	CACTTGTCCGTCGGGTAAT
*TMV-CP*	---------	ATTGTCATCAGCGTGGGC	TGCTGTGACTAGCGGGTCTA
*AtPR1*	AT2G14610	AATGCTCAAGATAGCCCACAAG	AATAAGTCACCGCTACCCCAG
*AtPDF1.2*	AT5G44420	TCACCCTTATCTTCGCTGCTC	ATGTCCCACTTGGCTTCTCG
