# Supplementary material for: Chitosan oligosaccharide induces resistance to Tobacco mosaic virus in Arabidopsis via the salicylic acid-mediated signalling pathway
Source: Sci Rep. 2016 May 18;6:26144. doi: 10.1038/srep26144 (PMC4870575; doi:10.1038/srep26144)
Supplement: Supplementary Information [file srep26144-s1.pdf]

Supplementary Information

Chitosan oligosaccharide induces resistance to *Tobacco mosaic virus* in *Arabidopsis* via salicylic acid-mediated signal pathway

Xiaochen Jia[a, b,+], Qingshan Meng[a,c,+], Haihong Zeng[a,d], Wenxia Wang[a], Heng Yin[a, *]

[a] Liaoning Provincial Key Laboratory of Carbohydrates, Dalian Institute of Chemical Physics, Chinese Academy of Sciences, Dalian 116023, China

[b] University of Chinese Academy of Sciences, Beijing, 100049, China

[c] College of Life Science, Dalian University of Technology, Dalian 116023, China

[d] College of Food Science and Engineering, Dalian Ocean University, Dalian 116023, China

[+] These authors contributed equally to this work

*Corresponding author at: Liaoning Provincial Key Laboratory of Carbohydrates, Dalian Institute of Chemical Physics, Chinese Academy of Sciences, Dalian 116023, China.

E-mail address: yinheng@dicp.ac.cn (H. Yin).

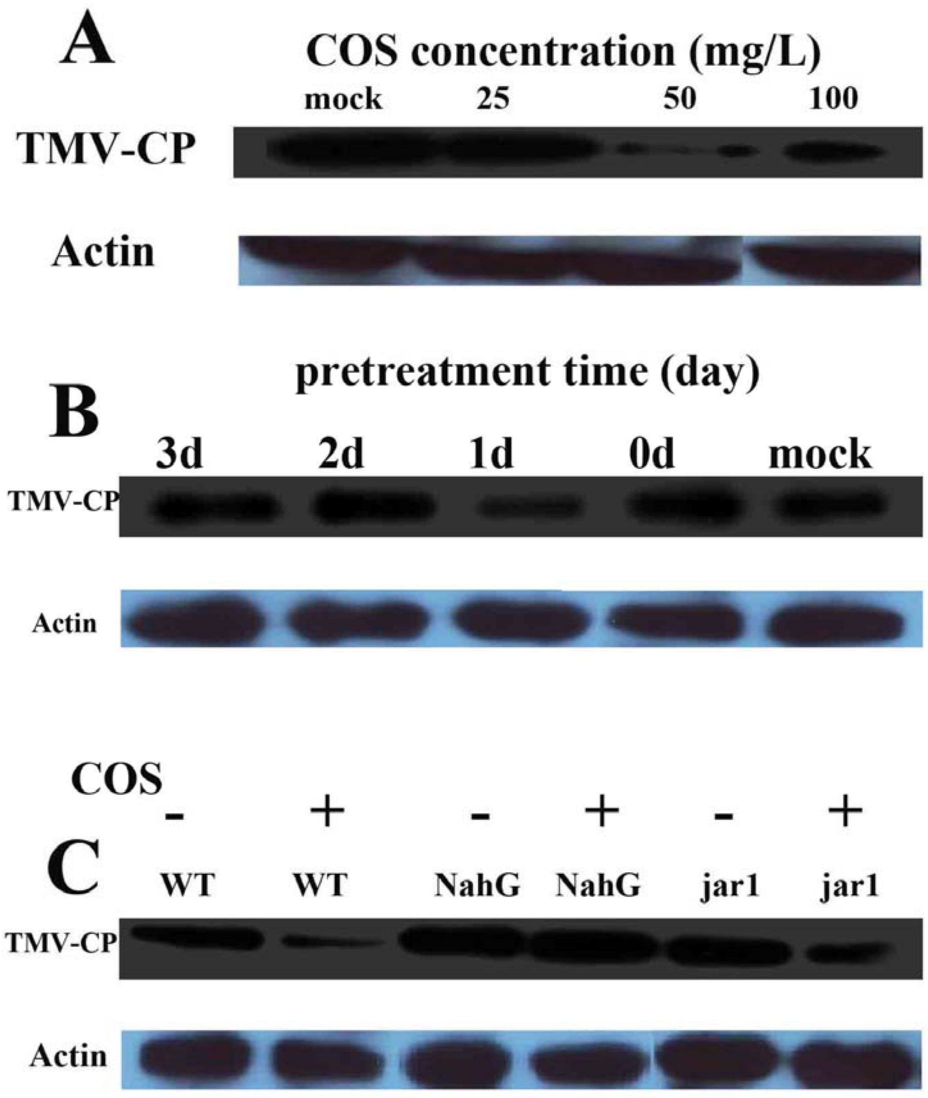

Figure S1. The full-length western-blots in Fig.1 and Fig.3. A for Fig.1A; B for Fig.1B; C for Fig.3A, These were the representative experiment and were independently repeated three times using different samples from independent treatment. The same amount of protein was loaded on SDS-PAGE gels, detected by anti-plant actin antibody, which served as the protein loading control. The detection of TMV-CP and loading control were carried out under the same experimental conditions.
